# Long-term patency and clinical outcome of the transjugular intrahepatic portosystemic shunt using the expanded polytetrafluoroethylene stent-graft

**DOI:** 10.1371/journal.pone.0212658

**Published:** 2019-02-27

**Authors:** Xuefeng Luo, Ming Zhao, Xiaoze Wang, Mingshan Jiang, Jiaze Yu, Xiao Li, Li Yang

**Affiliations:** 1 Department of Gastroenterology and Hepatology, West China Hospital, Sichuan University, Chengdu, China; 2 Department of interventional radiology, National Cancer Center/Cancer Hospital, Chinese Academy of Medical Science and Peking Union Medical College, Beijing, China; Goethe-Universitat Frankfurt am Main, GERMANY

## Abstract

**Background:**

Transjugular intrahepatic portosystemic shunt (TIPS) creation is an established treatment option to management the complications of portal hypertension. Recent data on the long-term outcomes of TIPS are scarce.

**Materials and methods:**

In this single-institution retrospective study, 495 patients underwent TIPS with the Fluency stent-grafts between December 2011 and June 2015 were evaluated. The cumulative rates of TIPS dysfunction, hepatic encephalopathy (HE), survival, and variceal rebleeding were determined using the Kaplan–Meier method. Cox regression analysis was used to assess the parameters on TIPS patency, occurrence of HE and all-cause mortality.

**Results:**

Technical success was 98.2%. TIPS-related complications occurred in 67 patients (13.5%) during the index hospital stay. TIPS creation resulted in an immediate decrease in mean portosystemic pressure gradient from 23.4 ± 7.1 mmHg to 7.6 ± 3.5 mmHg. The median follow-up period was 649 days. Primary TIPS patency rates were 93%, and 75.9% at 1 and 3 years, respectively. Previous splenectomy was associated with a higher risk of TPS dysfunction. The cumulative survival rates were 93.4% and 77.2% at 1 and 3 years, respectively. The 1- and 3-year probability of remaining free of variceal bleeding rates were 94.2% and 71.4%, respectively.

**Conclusions:**

This retrospective single-center experience with TIPS using the Fluency stent-grafts demonstrates good long-term patency and favorable good clinical results. Previous splenectomy strongly predicts shunt dysfunction.

## Introduction

Transjugular intrahepatic portosystemic shunt (TIPS) is an established, minimally invasive intervention for the treatment of the complications of portal hypertension such as variceal bleeding and refractory ascites [1, 2]. TIPS reduces portal pressure through establishing a shunt tract from the portal venous system to the hepatic venous system using bare or stent-grafts. Since the dedicated Viatorr expanded polytetrafluoroethylene (ePTFE) stent-grafts for TIPS became commercially available, shunt patency has greatly improved [3–5]. However, a recent retrospective study showed that the primary TIPS patency rate was not so satisfied (74% at 2 years and 50% at 6 years) [6]. Patients with a dedicated TIPS stent-graft may still require long-term clinical follow-up and shunt revision, which carry additional medical cost and procedural risks.

ePTFE–covered Fluency Plus stent-grafts have been widely used for TIPS procedures in China due to long-term lack of the Viatorr stent-graft (W.L. Gore & Associates, Flagstaff, AZ, USA). The Viatorr stent-graft has a 2-cm uncovered segment at the portal end and the Fluency stent-graft has a 2-mm uncovered portion at each end. Considering the different structures and product characteristics, it is meaningful to investigate the efficacy of the Fluency stent-graft. We report our experience using TIPS with the ePTFE-covered Fluency stent-graft TIPS, specifically addressing long-term patency and its clinical outcomes.

## Materials and methods

### Patient selection

This retrospective study has been approved by the local ethics committee of West China Hospital and is in accordance with the Declaration of Helsinki. Between December 2011 and June 2015, 571 consecutive patients with symptomatic portal hypertension due to liver cirrhosis were referred for de novo TIPS creation using ePTFE-covered stent-grafts in a single major metropolitan hospital. Liver cirrhosis was diagnosed based on medical history, lab examinations, imaging findings, and/or biopsy. Forty-one patients were lost to follow-up during the first month after the TIPS procedure, 23 patients with hepatic cell carcinoma (HCC), one patient with prior liver transplantation and 10 patients in whom TIPS was not technically successfully were excluded, leaving 495 patients for the final analysis.

### TIPS technique

The TIPS procedures were performed by four interventional radiologists and all of the procedures were supervised by the same doctor experienced in performing TIPS. Generally, a right internal jugular vein was used to advance a 10F sheath into the right atrium and the right atrium pressure was measured using a 5F pigtail catheter (Cook, Bloomington, USA). Then a TIPS set (Cook, Bloomington, USA) was introduced into the hepatic vein. Direct intrahepatic portocaval shunt (DIPS) was performed via the inferior vena cava in patients with complete occlusion of the hepatic veins. Contrast-enhanced computed tomography (CT) prior TIPS procedure was used to document portal vein patency and establish the relative anatomy between the starting point (hepatic vein) and end point (portal vein). The angle of the Colapinto metal cannula was manually adjusted and a needle inserted through the 5F catheter is then used to puncture the liver from a central portion of the hepatic vein and enter the right or left portal vein trunk to ensure a smooth shunt angulation. If the initial attempts (usually less than 5 punctures) failed to gain access into the portal vein, post-anterior and lateral indirect portography via the superior mesenteric artery (SMA) was applied to provide a target for needle pass. Specifically, a total of 24 ml contrast medium (Omnipaque, 300 mg/ml) each time was injected into the SMA through a catheter by a power injector (350 psi, Mark V ProVis injection System; Medrad) at a rate of 4 ml/s. Exposure time was extended until the intrahepatic portal vein was visualized. When blood is easily aspirated through the catheter, a small amount of contrast media is gently injected under fluoroscopy guidance to confirm the portal vein puncture. Then the direct portography was performed with a catheter adjacent to the spleen hilum to illustrate the whole portal venous system. Portal vein pressure was measured with a 5F pigtail catheter located at the confluence of the superior mesenteric and splenic vein. The intrahepatic parenchymal tract was predilated with an 8 x 60 mm angioplasty balloon (Cordis, Roden, the Netherlands), followed by the insertion of an 8- or 10-mm Fluency ePTFE-covered stent-graft (Bard, Murray Hill, USA). The length between the hepatic vein and the portal vein was roughly estimated by the length of the waist of the 6-cm long balloon. In fact, a 6-cm length balloon could be used to partially stimulate the configuration of a 6-cm length stent-graft after deployment. The 6-cm or 8-cm Fluency stent-graft was chosen accordingly. A second bare stent (Cordis, Roden, the Netherlands) or Fluency stent-graft would be inserted coaxially if the first stent-graft could not maintain sufficient intrastent blood flow. The portosystemic pressure gradient (PSG) was measured as the difference between the portal vein pressure and the right atrial pressure. A post-dilation was performed if the PSG was not reduced to below target threshold (12mmHg). In general, gastrorenal shunt or persistent visualized varices on post-TIPS portography were embolized using metal coil (Cook, Bloomington, USA) or plug (Huayi shengjie, Beijing, China). Glue (Guangzhou baiyun, Guangdong, China) was used as a supplementary embolic material after the blood flow in the varices was slow down by the coil or plug. Following TIPS procedures, low molecular heparin was given to all the patients for 3–5 days, except those with suspected intraoperative hemorrhage. Warfarin was prescribed only in patients with portal vein thrombosis (PVT) or underlying coagulation disorders. Antiplatelet therapy was not routinely used. Prophylactic antibiotic treatment was not given routinely.

### Follow-up and definition

Patients were monitored for 72 h after TIPS procedure and were then followed until liver transplantation, death, or loss to follow-up. Patients were evaluated at 1, 3, and 6 months after discharge and every 6 months with full clinical, hematological, biochemical, and Doppler ultrasonography (US) assessment. Abdominal contrast-enhanced CT and endoscopy were performed annually. TIPS dysfunction was suspected at Doppler US if intrastent flow velocity was less than 60 cm/sec or more than 180 cm/sec or if an absence of blood flow within the stent-graft occurred, as well as the recurrence of symptoms, such as variceal bleeding or ascites. The direct portography via the initial TIPS and manometry was not carried out routinely, but only in patients with suspected dysfunctional TIPS. Variceal rebleeding was defined as a single episode of clinically significant rebleeding (recurrent melena or hematemesis resulting in hospital admission, blood transfusion, a drop of hemoglobin by 3g/L, or death within 6 weeks) from portal hypertensive sources.

### Statistical analysis

Distribution of variables was assessed using the Kolgomorov-Smirnov test. All continuous variables distributed normally were expressed as mean value ± standard deviation, otherwise as median and interquartile range. The categorical variables were expressed as absolute and percentage values. The cumulative rates of TIPS dysfunction, hepatic encephalopathy (HE), survival, and variceal rebleeding were determined using the Kaplan–Meier analysis, and the comparison between groups via the log-rank test. Risk factors with *P* < 0.05 in the univariate analysis were included in a multivariate Cox regression analysis by the forward stepwise method. Bonferroni correction was applied to address Type 1 error. Pearson and Spearman correlation was used to assess the correlation between risk factors. If the risk factors were correlated, then only one of these factors was selected for the multivariate analysis. Hazard ratios (HRs) with 95% confidence intervals (CIs) were calculated. The component of Child–Pugh and Model for End Stage Liver Disease (MELD) score was not included in the univariate analysis. The differences were considered statistically significant at a *P* value of < 0.05. The SPSS 22.0 statistical package (SPSS Inc., Chicago, IL, USA) was used for data analysis.

## Results

### Patient characteristics

Patient characteristics at the time of TIPS are presented in Table 1. The median age of selected patients was 51 years (range, 15–83 years), and 65.7% were male and 34.3% were female. Hepatitis B cirrhosis and alcoholic cirrhosis were identified in 59.6% and 18.4% of our patients, respectively. The most common indication for TIPS creation was variceal bleeding (n = 436, 88.1%), and most patients had Child–Pugh B disease (n = 281, 56.8%). The median Child–Pugh and MELD scores before TIPS placement were 7 and 10 respectively. PVT coexisted in 99 patients (20%). The median follow-up period was 649 days.

**Table 1:** Baseline characteristics of subjects.

Number of patients	N = 495
Age, years, median (range)	51 (44–61)
Gender (male/female)	325/170
Etiology of liver disease [n(%)]	
Hepatitis B cirrhosis	295 (59.6%)
Alcoholic cirrhosis	91 (18.4%)
Primary biliary cirrhosis	34 (6.9%)
Hepatitis C cirrhosis	19 (3.8%)
Autoimmune hepatitis	13 (2.6%)
Schistosoma cirrhosis	12 (2.4%)
Other	31 (6.3%)
Indication [n(%)]	
Variceal bleeding	436 (88.1%)
Refractory ascites	36 (7.3%)
Others	23 (4.6%)
Laboratory values	
Bilirubin (μmol/L)	19.4 (14.5–27.5)
Albumin (g/L)	32.5 ± 5.6
BUN (μmol/L)	5 (3.73–6.65)
Creatinine (μmol/L)	69.9 (59–81)
INR	1.27 (1.18–1.39)
Platelet (10^9^/L)	63 (43–91)
White blood cell (10^9^/L)	3.32 (2.23–4.47)
Encephalopathy [n(%)]	9 (1.8%)
Ascites (no/small/medium/large)	174/146/69/106
Portal vein thrombosis [n(%)]	99 (20%)
Previous splenectomy [n(%)]	47 (9.5%)
Child-Pugh grade [n(%)]	
A	172 (34.7%)
B	281 (56.8%)
C	42 (8.5%)
Child-pugh score	7 (6–8)
MELD score	10 (9–12)
Follow-up period, days, median (range)	649 (426–1033)

Abbreviations: BUN, blood urea nitrogen; INR, international normalized ratio; MELD, Model for End Stage Liver Disease.

### TIPS procedure

Portal vein visualization using indirect portography via SMA was required in 65 patients (13.1%). In 474 patients (95.8%), the TIPS was created via the right or middle hepatic vein, whereas in the other 21 patients (4.2%), the DIPS was performed. The TIPS was established through the right portal branch in 338 patients (68.3%), the left portal branch in 109 patients (22%), the bifurcation in 46 patients (9.3%), and the main portal vein in 2 patients (0.4%). The stent-graft lengths used were 6 cm (n = 415, 83.8%) and 8 cm (n = 80, 16.2%). 8-mm shunt was established in 57 patients (11.5%). PSG was reduced from 23.4 ± 7.1 mmHg to 7.6 ± 3.5 mmHg (*P* <0.001).

TIPS-related complications occurred in 67 patients (13.5%) during the index hospital stay. Post-TIPS infection occurred in 60 patients, of whom 11 developed septicemia by a blood culture analysis. Four patients were tested positive for Escherichia coli, two patients for Klebsiella pneumonia and the remaining five patients for Burkholderia cepacia, Gemella morbillorunm, Streptpcpccus cristatus, Citrobacter feundii and Aeromonas hydrophila, respectively. Displacement of metal coils occurred in four patients and unintentional flow of the mixture of glue and iodinated oil into the pulmonary arteries leading to pulmonary embolization was observed in one patient; none of these patients presented clinical symptoms. Four patients experienced intra-abdominal bleeding, and one experienced bile duct injury and biliary hemorrhage. Each patient responded well to the conservative therapy without the requirement of surgery. One patient died during the procedure as a result of cardiac arrest, possibly due to the underlying heart disease.

### TIPS dysfunction

**Fig 1:**
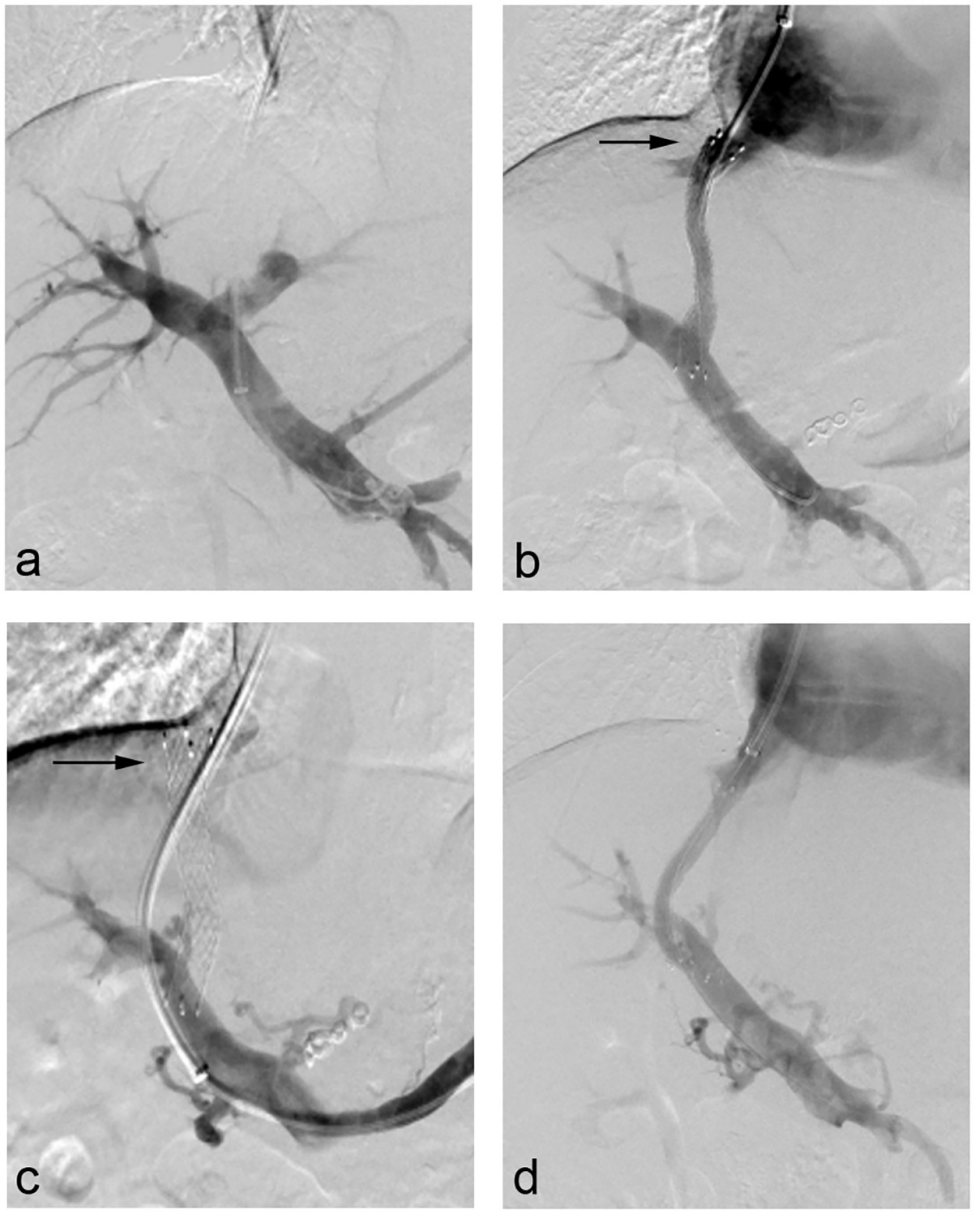
Shunt dysfunction occurred in a 56-year-old man 14 months after TIPS insertion. (a) Preliminary portogram performed after the left portal vein was accessed. The portosystemic gradient (PSG) measured 31 mmHg. (b) Portogram after deploying Fluency stent (C.R. Bard) showing a patent TIPS with the distal end at the hepatocaval junction (arrow) and the proximal end in the main portal vein. The PSG was reduced to 10 mmHg. (c) Digitally subtracted image demonstrates that the original shunt was occluded and the superior margin (arrow) of the stent penetrated into the hepatic vein wall. The PSG measured 28 mmHg. (d) Since the original shunt was difficult to catheterize, a parallel TIPS was placed via the right portal vein. Then the PSG decreased to 8 mmHg.

**Fig 2:**
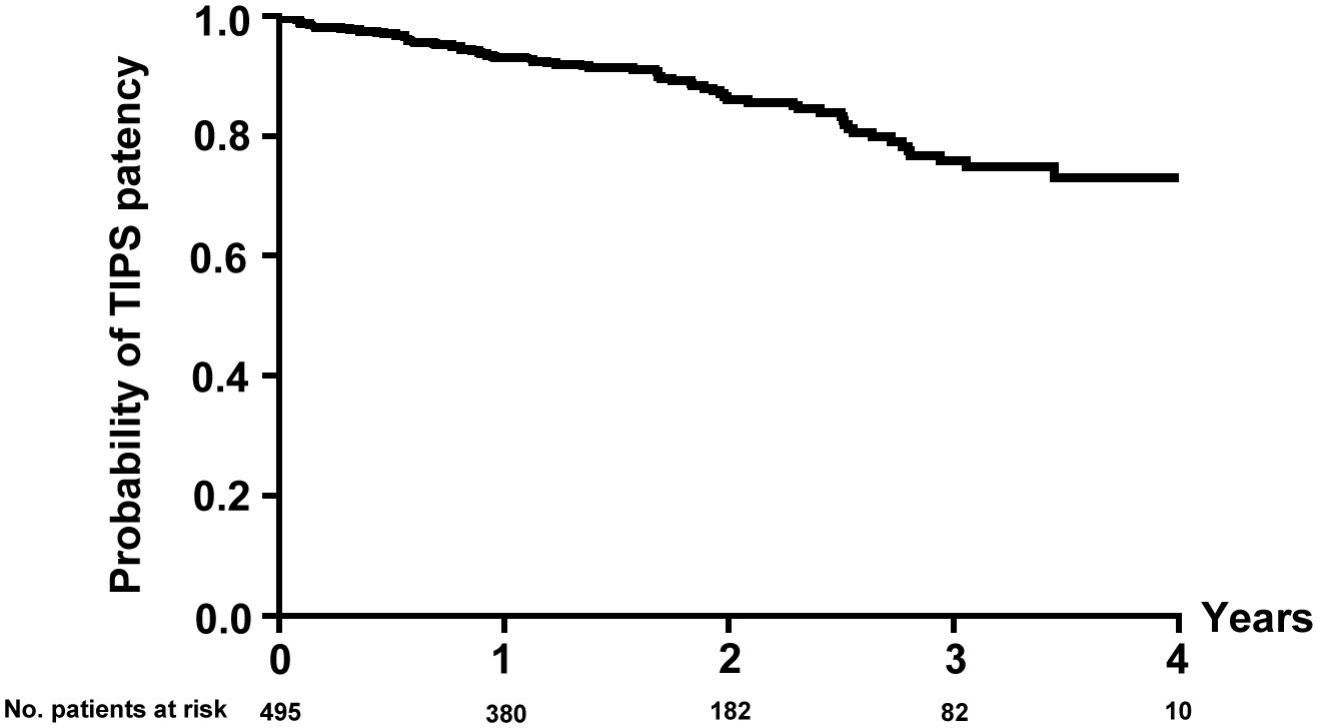
Probability of TIPS patency.

TIPS dysfunction occurred in 67 out of 495 patients (13.5%) during the whole follow-up period. Portography in the 37 patients who underwent shunt revision in our institution showed 18 complete shunt occlusions, 8 hepatic vein stenoses, 5 intrastent stenoses, and 6 portal vein stenoses. The change of stent-graft configuration was considered to be responsible for TIPS dysfunction in 18 out of 37 patients (48.6%) (Fig 1). Of these 37 patients, seven underwent shunt angioplasty and 12 required coaxial stent placement. In the other 18 subjects, the original TIPS could not be accessed or recanalization of the initial shunt required at least two stents, hence a parallel TIPS was established. Three patients needed a third shunt revision, each within 6 months after the second revision. The cumulative TIPS patency rates at 1 and 3 years were 93% and 75.9%, respectively (Fig 2). Of the variables assessed, a Cox univariate analysis revealed that previous splenectomy (HR 2.571; CI 95% 1.426–4.635; *P* = 0.012) was associated with TIPS dysfunction. (Table 2).

**Table 2:** Independent risk factors for shunt dysfunction.

Variable	Univariate analysis	
	HR (95% CI)	p value
Splenectomy	2.571 (1.426–4.635)	0.012*
Child-pugh score	0.873 (0.743–1.026)	0.1
TIPS via the left portal branch	1.943 (1.178–3.204)	0.054*
Number of stents	0.943 (0.494–1.801)	0.859
Stent length	0.856 (0.626–1.171)	0.332
Stent diameter	2.266 (0.545–9.414)	0.260

* denote *P*-value adjusted by use of the Bonferroni correction

Abbreviations: HR, hazard ratio; CI, confidence interval; TIPS, transjugular intrahepatic portosystemic shunt.

### Hepatic encephalopathy

**Fig 3:**
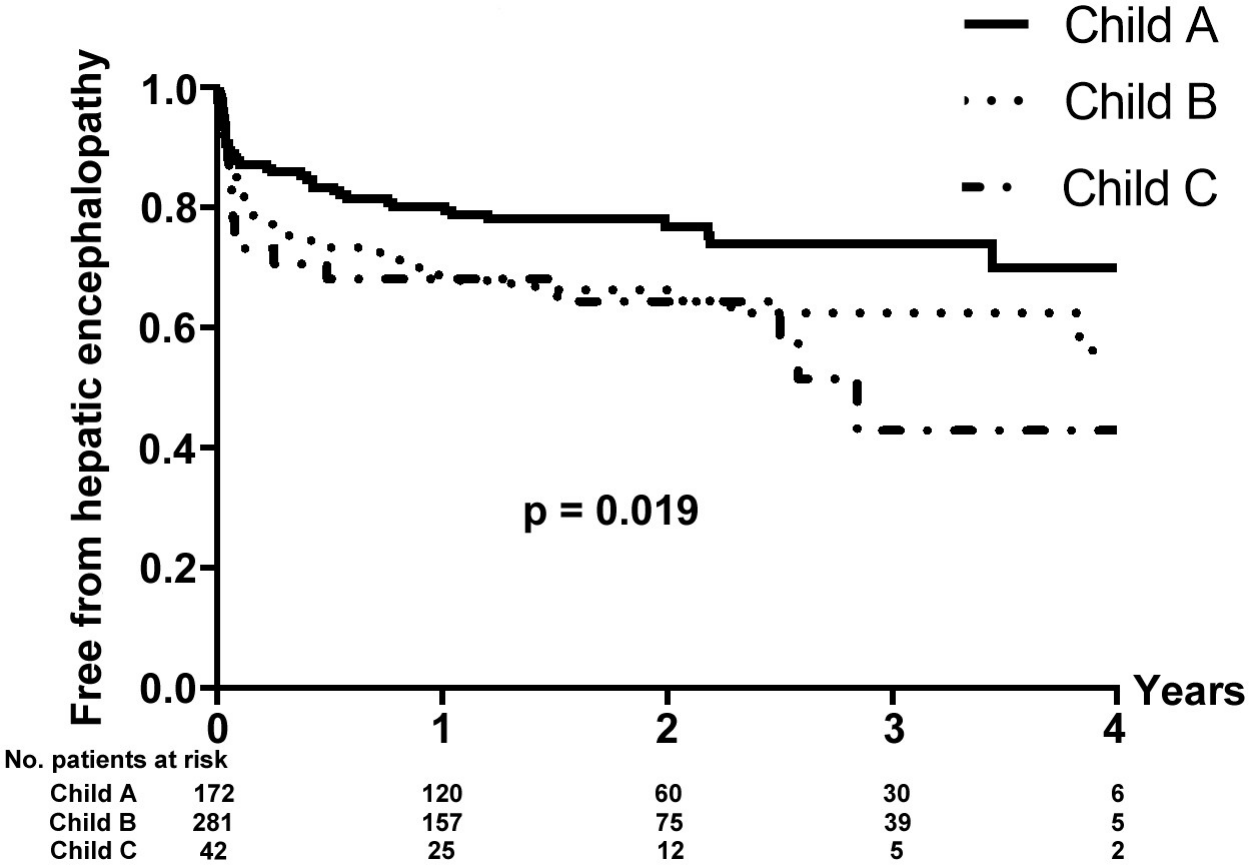
Probability of remaining free from hepatic encephalopathy in patients with different Child-pugh classes.

Four-hundred and sixty episodes of post-TIPS HE occurred in 151 out of 495 patients (30.5%), in whom 61 patients (40.4%) developed only one episode of HE during the follow-up. Refractory HE was observed in 18 patients (3.6%) of the cohort. The cumulative 1 and 3 years of free from HE rates were 72.7% and 64.6%, respectively. There was a significant difference of the cumulative rates of HE between patients according to Child-pugh Class (*P* = 0.019) (Fig 3). In the univariate analysis, only Child–Pugh score (HR, 1.169; 95% CI: 1.069–1.278; *P* = 0.009) was found to be associated with post-TIPS HE (Table 3).

**Table 3:** Independent risk factors for hepatic encephalopathy.

Variable	Univariate analysis	
	HR (95% CI)	p value
Age > 65	1.598 (1.065–2.397)	0.207*
Sex (Male / Female)	0.919 (0.655–1.290)	0.627
Indications of TIPS (VB / Non-VB)	0.698 (0.440–1.108)	1.108
Previous encephalopathy	1.334 (0.493–3.605)	0.570
PVT	1.014 (0.680–1.513)	0.945
BUN (μmol/L)	1.032 (1.001–1.065)	0.378*
White blood cell (10^9^/L)	0.996 (0.928–1.068)	0.902
Stent diameter	0.959 (0.570–1.616)	0.876
Child-pugh score	1.169 (1.069–1.278)	0.009*

* denote *P*-value adjusted by use of the Bonferroni correction

Abbreviations: HR, hazard ratio; CI, confidence interval; TIPS, transjugular intrahepatic portosystemic shunt; VB, variceal bleeding; PVT, portal vein thrombosis; BUN, blood Urea Nitrogen.

### Mortality

Seventy-three patients (14.7%) died after a median of 405 days (range, 0–1319 days). One patient died during the TIPS procedure due to heart arrest. Of the other 72 patients, 29 died of liver failure, 11 of multiple organ dysfunction syndrome, 8 of gastrointestinal bleeding, 6 of sepsis, 6 of HCC, 3 of renal failure, 1 of myocardial infarction, 1 of cerebral hemorrhage, and the cause was unknown in 7 patients. The cumulative survival rates were 93.4% and 77.2% at 1 and 3 years, respectively. There was no significant difference of median survival between patients with and without post-TIPS HE (644 vs. 676 days, *P* = 0.972). Patients in Child–Pugh class C had significantly higher cumulative mortality, with a 4-year survival rate of 25.1%, compared with 68.3% for patients in Child–Pugh class B and 89.4% for patients in Child–Pugh class A (*P* < 0.001). Predictors associated with a fatal outcome in the univariate analysis were age > 65, higher baseline blood urea nitrogen (BUN), and higher Child–Pugh and MELD scores. Including all significant variables at the univariate analysis, on multivariate analysis, age > 65 (HR 2.094; CI 95% 1.247–3.516; *P* = 0.005), a higher Child–Pugh score (HR 1.34; CI 95% 1.247–1. 516; *P* < 0.001), and a higher baseline BUN (HR 1.069; CI 95% 1.034–1.106; *P* < 0.001) were associated with the increased risk of mortality (Table 4).

**Table 4:** Independent risk factors for mortality.

Variable	Univariate analysis		Multivariate analysis	
	HR (95% CI)	p value	HR (95% CI)	p value
Age > 65	2.427 (1.450–4.063)	0.007*	2.094 (1.247–3.516)	0.015*
Sex (Male / Female)	0.928 (0.734–1.173)	0.532		
PVT	1.268 (0.745–2.160)	0.382		
BUN (μmol/L)	1.088 (1.057–1.120)	< 0.001	1.069 (1.034–1.106)	< 0.001
White blood cell (10^9^/L)	1.019 (0.924–1.124)	0.706		
Stent diameter	2.661 (0.645–10.979)	0.176		
Child-pugh score	1.431 (1.271–1.610)	< 0.001	1.34 (1.247–1. 516)	< 0.001

* denote *P*-value adjusted by use of the Bonferroni correction

Abbreviations: HR, hazard ratio; CI, confidence interval; PVT, portal vein thrombosis; BUN, blood Urea Nitrogen.

### Variceal rebleeding

**Fig 4:**
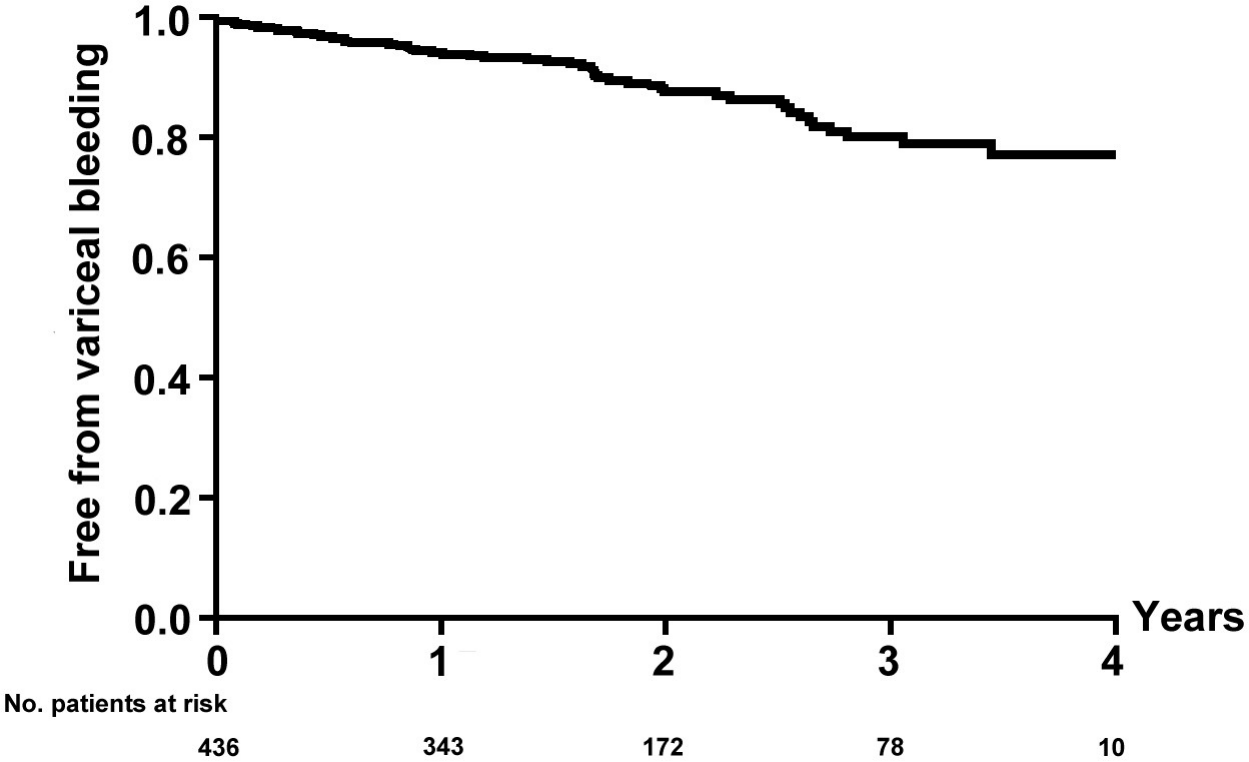
Probability of remaining free from variceal bleeding.

The indications of the 436 patients selected for analysis were acute uncontrolled variceal bleeding in 24 patients and a second prophylactic of variceal rebleeding in another 412 patients. Active gastrointestinal bleeding ceased immediately following TIPS placement in all 24 patients. During follow-up, variceal rebleeding occurred in 53 patients (12.2%). The 1- and 3-year probability of remaining free of variceal bleeding rates were 94.2% and 81.4%, respectively (Fig 4). There was no significant difference of variceal rebleeding rates between patients in different Child–Pugh classes (*P* = 0.255). Three patients died immediately after massive gastrointestinal rebleeding before TIPS portography could take place. TIPS were considered patent in 7 patients. The reasons for variceal rebleeding in these seven patients were as follows: (1) gastric varices (GVs) were not embolized (n = 4), GVs were not embolized thoroughly (n = 2), and post-TIPS PSG higher than 12mmHg (n = 1). The remaining 43 patients who rebled as a result of TIPS dysfunction were managed as follows: 23 had TIPS revision, 2 underwent splenectomy and esophageal transection, 2 required liver transplantation, and 16 responded well to endoscopic and/or drug therapy. There were 31 additional events of nonvariceal gastrointestinal hemorrhage with the reasons as follows: (1) peptic ulceration (n = 7); (2) coagulation disorders (n = 2); (3) Mallory–Weiss tear (n = 2); (4) esophagitis (n = 1); (5) small intestinal malformation (n = 1); (6) colonic hemorrhage (n = 1); and (7) hemobilia (n = 1). There were 16 recorded rebleeding episodes where the source of the bleed was not identified at endoscopy or the patient did not have an endoscopic examination.

## Discussion

Our studyprovides an opportunity to evaluate long-term shunt patency and clinical outcomes of TIPS creation with the use of ePTFE-covered Fluency stent-grafts. The observed TIPS patency rates were favorable and consistent with ranges found in earlier investigations evaluating TIPS creation with Viatorr stent-grafts [4, 6]. Previous splenectomy appeared to be a risk factor of TIPS dysfunction. Likewise, the present series demonstrates a high rate of technical success, acceptable post-TIPS complications, and fairly low mortality.

Long-term TIPS patency rates in our study were favorable. Our study differed from the previous reports in several aspects, which may partly explain our better results. First, we began to use Fluency stent-grafts during TIPS procedure since 2011, much later than the clinical application of other ePTFE stent-grafts. The underlying mechanisms of TIPS dysfunction using ePTFE stent-grafts have been revealed thoroughly [7, 8]. The standard protocols of the TIPS procedure we adopted in our hospital have been updated and improved on the basis of these findings [1, 2, 9]. A second stent was deployed in 36 patients due to the stent shortage in the haptic vein, which is the most common reason for shunt dysfunction. Second, only the patients with abnormal intrastent flow velocity and the recurrence of complications of portal hypertension who were suspected to have a dysfunctional shunt and were advised to undergo direct portography. This management strategy may underestimate the actual shunt dysfunction rate. Even so, we believe that portal hypertension-related symptom recurrence should be included in the evaluation of TIPS function.

Of 37 patients who underwent shunt revision, 18 patients (48.6%) had complete shunt occlusion, which is significantly higher than in previous case series (16%) by Weber et al[6]. Failure to detect shunt stenosis before the complete occlusion could increase the difficulties of shunt revision and the risk of recurrence of fatal complications of portal hypertension. Although we adopted close Doppler US surveillance, the actual efficiency could be negatively influenced by the patient compliance, shortage of trained sonographers in basic hospitals, and lack of contrast-enhanced US. Though Doppler US is the most cost-effective method for the assessment of TIPS patency, the interobserver inconsistency may be high as a result of the difference in the sonographer’s experience and different equipment. Appropriate thresholds of the absolute value of intrastent blood flow velocity and the change of blood flow velocity compared with pre-TIPS parameters need to be established and validated[1].

Notably, nearly half (48.6%) of the shunt dysfunction was associated with the alteration of shunt configuration. This is possibly attributed to the rigid structure and radial forces of Fluency stent-grafts. The outline of the stents became straight gradually after the deployment instead of maintaining the original arc shape within the hepatic parenchyma. Even if the superior margin of the TIPS was extended to the junction of the hepatic vein and IVC, it may direct toward the superior wall of the hepatic vein in a perpendicular fashion, eventually resulting in compromised blood and shunt dysfunction. It has been widely recommended that the covered portion of a dedicated Viatorr stent-graft should be extended to the orifice of the hepatic vein at the IVC [2, 9]. Since the Fluency stent-graft straightens into its nominal configuration over time, the strategy of the deployment of this device, such as generic stent-graft/bare stent combination, needed to be further evaluated and validated.

One of the major limitations of TIPS is the development of HE, which occurred in 30.5% of our patients. In line with earlier studies, poorer liver function was associated with an increased risk of post-TIPS HE in our study. One of the most overriding concerns with the Fluency stent-graft is the lack of 2 cm uncovered segment in the portal end, which may block the intrahepatic portal branches and decrease portal perfusion theoretically. However, the effect of Fluency stent-graft on the post-TIPS portal perfusion and the incidence of HE rate has not been addressed properly. The reported post-TIPS HE rates using Fluency stent-grafts varied from 15.3% to 31.3% in different studies[10–14]. Although we reported a much higher incidence of HE than in some other studies, it was worthy noted that of the 151 patients with post-TIPS HE, 61 (40.4%) patients experienced only one episode of HE and merely 18 patients had refractory HE.

Mortality in the TIPS population remains high despite the complications of portal hypertension in the vast majority, with most deaths due to liver failure. The explanations for relatively better survival in our series may include the following aspects: first, most of the patients in this study had relatively better liver function (a mean Child–Pugh score of 7.3 and a mean MELD score of 10.8). Second, our patients had fewer complicating diseases. Third, there were only 36 TIPS procedures due to refractory ascites, which indicated poorer outcomes. Patients with Child–Pugh C class liver disease had a far worse outcome than patients with A and B classes. Our findings corroborate results of other investigations that established the value of the advanced age (> 65), a higher Child–Pugh score, and BUN as predictors of survival in patients undergoing TIPS creation[15–19]. Furthermore, the results of the present study revealed a lower age threshold (65 y) for an increased risk of mortality compared with previously identified age cutoffs. Our results also confirmed that a higher Child–Pugh score predicted poor survival following TIPS creation[20]. This suggests initial liver function being the vital prognostic factor to predict survival after TIPS.

Consistent with recently published prospective RCTs, the present study confirmed that TIPS creation could significantly decrease the incidence of variceal rebleeding [21, 22]. In most TIPS cases, recurrent bleeding episodes were attributed to shunt dysfunction instead of Child–Pugh class. Notably, TIPS were not deemed as dysfunctional in 7 patients in spite of reoccurrence of portal hypertension-related gastrointestinal hemorrhage. Prevention of variceal bleeding is not solely dependent on portal venous system decompression. Appropriate management of varices during the TIPS procedure should be established in consideration of endoscopic classification of varices, post-TIPS PSG, the presence of porto-systemic shunts, and characteristics of different embolization materials. In addition, our study found that nonvariceal bleeding in patients with TIPS is not uncommon (7.1%).

The present investigation is limited by its single center and retrospective design without a control group. Nevertheless, our institution is a referral center for hepatic diseases and one of the largest hospitals in China. Secondly, it is difficult to make every patient comply with our post-TIPS surveillance requirement and perform face to face evaluation. We gave out detailed contact information of our department and performed follow-up by telephone regularly. In the meantime, 41 patients who lost to follow-up during the first month after the TIPS procedure were excluded from the analysis to minimize bias. Third, the effect of the covered segment of the Fluency stent-graft in the portal vein on the intrahepatic portal hemodynamic was not evaluated, because the sequential follow-up CT data was not adequate. Even so, the present study could provide valuable information with regards to the use of Fluency stent-graft for TIPS creation.

## Conclusion

This is the largest study using Fluency stent-grafts and confirms the efficacy of TIPS in the management of portal hypertension and favorable long-term patency compared with other ePTFE stent-grafts in a real-life setting. Previous splenectomy is associated with an increased risk of shunt dysfunction. Since Viatorr stent-grafts recently became available in China, future studies are required to compare the patency rates and the cost effectiveness between these two types of stent-grafts.

## Supporting information

S1 Checklist (DOC)

S1 Dataset (XLSX)
